# Vector-borne helminths of dogs and humans in Europe

**DOI:** 10.1186/1756-3305-6-16

**Published:** 2013-01-16

**Authors:** Domenico Otranto, Filipe Dantas-Torres, Emanuele Brianti, Donato Traversa, Dusan Petrić, Claudio Genchi, Gioia Capelli

**Affiliations:** 1 Dipartimento di Medicina Veterinaria, Università degli Studi di Bari, Bari, Valenzano, Italy; 2 Departamento de Imunologia, Centro de Pesquisas Aggeu Magalhães (Fiocruz-PE), Recife, Pernambuco, Brazil; 3 Dipartimento di Sanità Pubblica Veterinaria, Università degli Studi di Messina, Messina, Italy; 4 Dipartimento di Scienze Biomediche Comparate, Università degli Studi di Teramo, Teramo, Italy; 5 Laboratory for Medical and Veterinary Entomology, Faculty of Agriculture, University of Novi Sad, Novi Sad, Serbia; 6 Dipartimento di Patologia Animale, Igiene e Sanità Pubblica Veterinaria, Università degli Studi di Milan, Milan, Italy; 7 Istituto Zooprofilattico Sperimentale delle Venezie, Legnaro, Padova, Italy

**Keywords:** Zoonosis, *Dirofilaria immitis*, *Dirofilaria repens*, *Onchocerca lupi*, *Cercopithifilaria*, *Thelazia callipaeda*, Europe, Risk, Mosquito, Tick, Vector, Treatment, Control

## Abstract

Presently, 45% of the total human population of Europe, as well as their domestic and companion animals, are exposed to the risk of vector-borne helminths (VBH) causing diseases. A plethora of intrinsic biological and extrinsic factors affect the relationship among helminths, vectors and animal hosts, in a constantly changing environment. Although canine dirofilarioses by *Dirofilaria immitis* and *Dirofilaria repens* are key examples of the success of VBH spreading into non-endemic areas, another example is represented by *Thelazia callipaeda* eyeworm, an emergent pathogen of dogs, cats and humans in several regions of Europe. The recent finding of *Onchocerca lupi* causing canine and human infestation in Europe and overseas renders the picture of VBH even more complicated. Similarly, tick-transmitted filarioids of the genus *Cercopithifilaria* infesting the skin of dogs were recently shown to be widespread in Europe. Although for most of the VBH above there is an increasing accumulation of research data on their distribution at national level, the overall impact of the diseases they cause in dogs and humans is not fully recognised in many aspects. This review investigates the reasons underlying the increasing trend in distribution of VBH in Europe and discusses the diagnostic and control strategies currently available. In addition, this article provides the authors’ opinion on some topics related to VBH that would deserve further scientific investigation.

## Introduction

A large number of vector-borne helminths (VBH) are prevalent in Europe, and some of them are of growing importance due to the significant level of disease they cause in dogs and humans [1-3]. Presently, 45% of the total human population of Europe, as well as their domestic and companion animals, are exposed to the risk of VBH [4]. A complex range of intrinsic biological factors (e.g., vectorial capacity, biting rates), extrinsic and environmental factors (e.g., climate, population movements and trade), affects the interactions between parasitic helminths, vectors and animals, including humans, rendering investigations on VBH a complex task. Indeed, the spreading process of VBH in previously non-endemic geographical areas has been primarily associated with the biology and ecology of the arthropod vectors and their capability to establish transmission cycles, maintaining the infestation in populations of susceptible hosts. Since the beginning of the millennium, many vectors have been introduced into Europe as a consequence of human demographics (e.g., the growth of cities), international movement of people (travellers and refugees), the smuggling of wildlife, the trade of animals and goods, such as used tires and ornamental plants [5]. For example, human activities have initiated the spread of invasive mosquito species and vector-borne diseases, and on-going globalization and increases in mean temperature may greatly extend the magnitude of this process [4].

The present article is focused on major VBH infesting dogs and humans. Among this diverse group of pathogens, *Dirofilaria immitis* and *Dirofilaria repens* (Spirurida, Onchocercidae) are probably the best known. Indeed, *D. immitis* has a severe impact on veterinary medicine, because of the heartworm disease threatening dogs and cats, whereas *D. repens*, causing subcutaneous infestation in dogs, is the main agent of human dirofilariosis. Although *Dirofilaria* spp. above represent key examples of the success of this group of parasites in spreading into non-endemic areas, over the last ten years other zoonotic helminths, such as *Thelazia callipaeda* eyeworm (Spirurida, Thelaziidae) have been accounted as emergent VBH of animals and humans in several Europe regions [5]. In addition, the puzzle become even more complicated to solve by the recent finding of a little known filarioid of dogs, i.e. *Onchocerca lupi* (Spirurida, Onchocercidae), which causes canine and human infestation in Europe and overseas. This nematode primarily induces nodular lesions under the conjunctiva and sclera of dogs and its biology and actual distribution remain for many aspects unknown to science. Adults of the less known *Acanthocheilonema reconditum* and *Acanthocheilonema dracunculoides* are beneath the subcutaneous tissues of the limbs and back of dogs. Recently, tick-transmitted filarioids of the genus *Cercopithifilaria* infesting the skin of dogs were shown to be surprisingly distributed in canine populations of Europe [6].

Although for many of the VBH above there is an increasing accumulation of information about their distribution at national level, the overall impact of diseases they cause in dogs and humans is not fully recognised in many aspects. This review aims at investigating the main reasons underlying the increasing trend in distribution of the most important VBH in Europe and to discuss the diagnostic and control strategies currently available. In addition, this article provides the authors’ opinion on some topics related to VBH that would deserve further scientific investigation.

## Review

### Old and emerging VBH of dogs and humans in Europe

**Figure 1:**
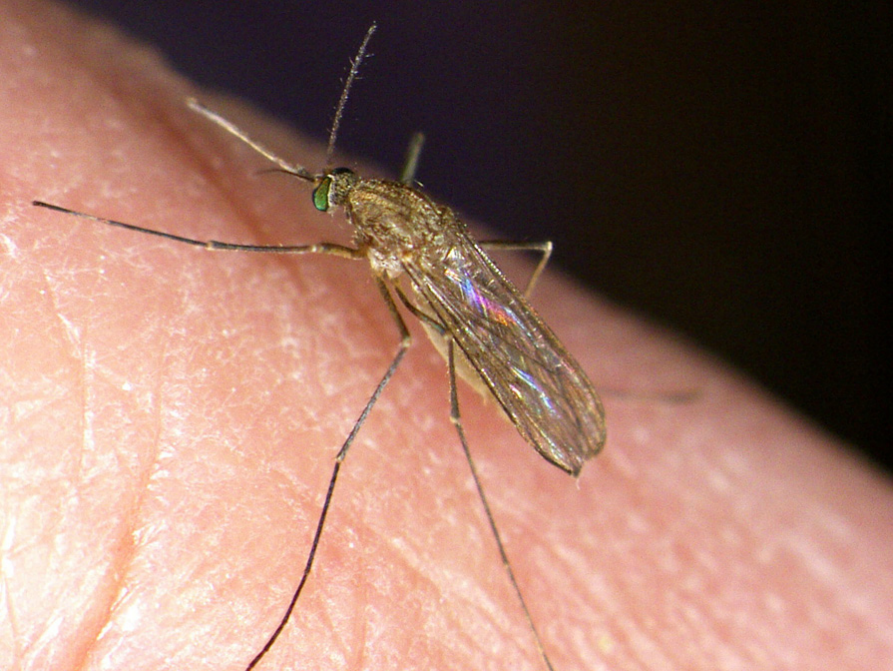
***Culex pipiens pipiens. ****Culex pipiens pipiens* feeding on a human host (Courtesy of Fabrizio Montarsi).

**Figure 2:**
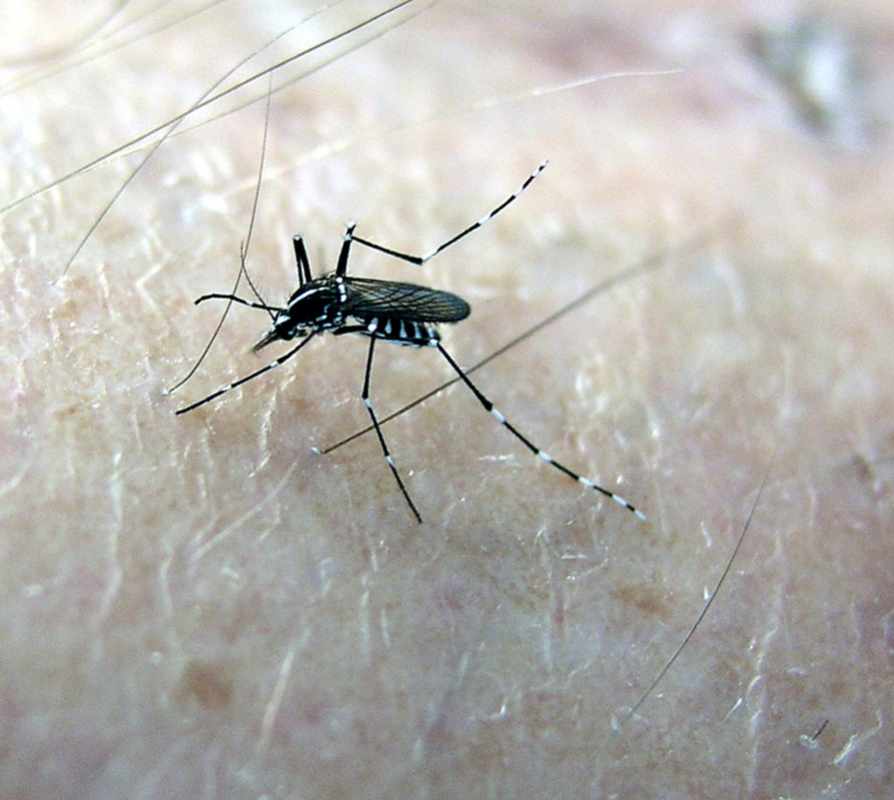
***Stegomyia albopicta. ****Stegomyia albopicta* feeding on a human host (Courtesy of Nediljko Landeka).

Dirofilarioses caused by filarioid nematodes of the genus *Dirofilaria* are transmitted at their third larval stage by bloodsucking mosquitoes primarily to dogs, although cases of human dirofilariosis are increasingly reported [1]. Adult *D. immitis* worms occur in the pulmonary arteries and right heart chambers, causing a severe condition, known as canine and feline heartworm disease, while *D. repens* is found mainly in subcutaneous tissues, causing subcutaneous dirofilariosis. *Dirofilaria* nematodes develop throughout five larval stages within the intermediate vector mosquito host (from embryo to infective L3 larva), and in the definitive vertebrate host (from L3 to the adult stage). The adult females of *D. immitis* and *D. repens* develop in 120–180 and 189–259 days, respectively, and release microfilariae into the blood of the definitive host [7]. The intermediate hosts are mosquitoes of the family Culicidae (e.g. *Anopheles, Aedimorphus*, *Armigeres, Ochlerotatus*, *Stegomyia*, *Culex*, *Coquillettidia* and *Mansonia*), with *Aedimorphus vexans* [*Aedes vexans*], *Culex pipiens pipiens* (Figure 1) and *Stegomyia albopicta* [*Ae. albopictus*] (Figure 2) being implicated as the main vectors of these worms in Europe [8]. *Dirofilaria repens* is able to grow under laboratory conditions in the same mosquito species and at the same temperature and humidity as *D. immitis*, with similar developmental time, from the microfilarial stage to the infective larva [7].

**Figure 3:**
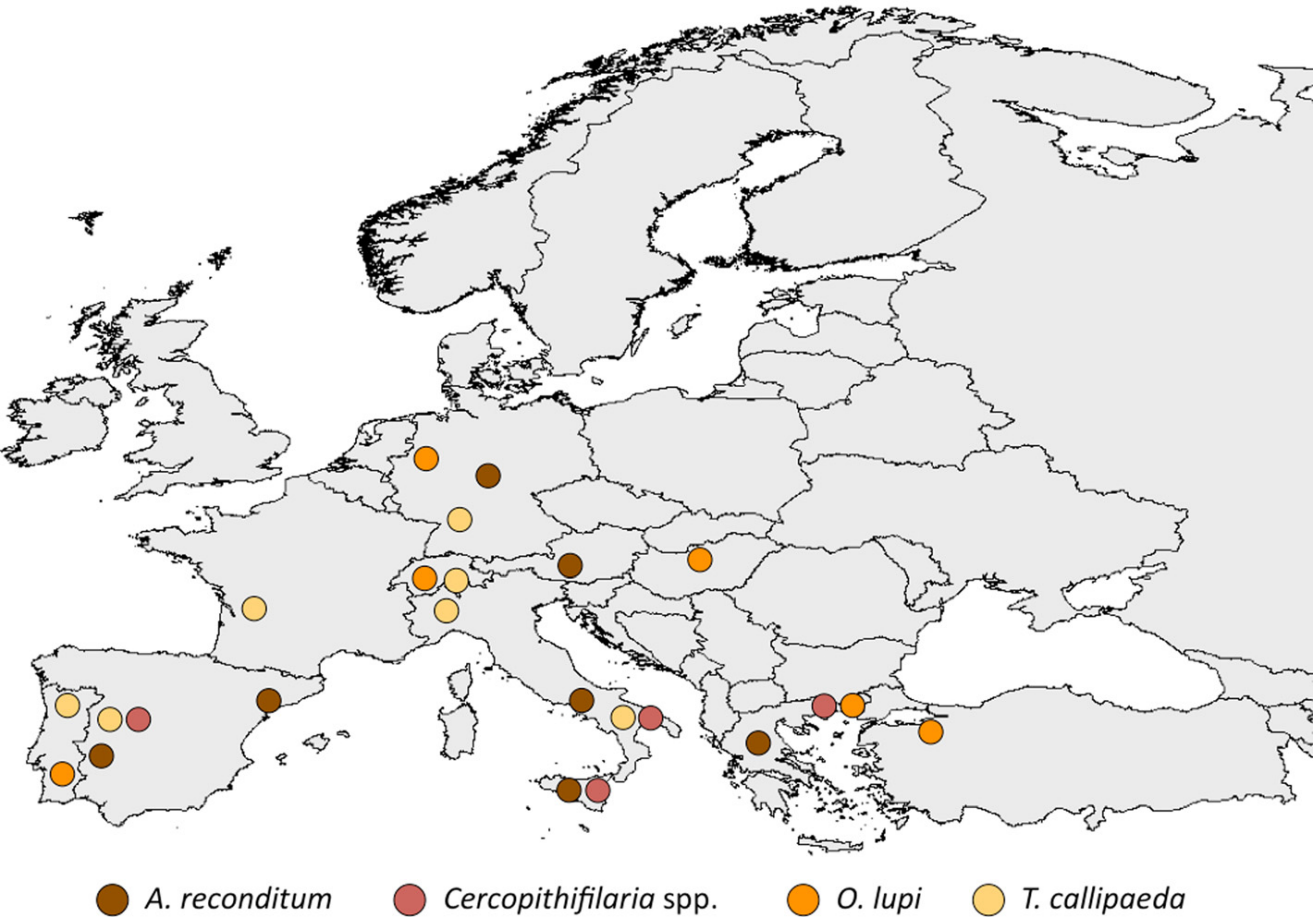
**Distribution of *Acanthocheilonema reconditum, Cercopithifilaria *spp., *Onchocerca lupi *and *Thelazia callipaeda *in Europe.** Map of Europe showing the distribution of *Acanthocheilonema reconditum, Cercopithifilaria* sp.1, *Onchocerca lupi* and *Thelazia callipaeda*.

*Acanthocheilonema reconditum* has a global distribution and, in many geographical areas of the Mediterranean Basin (Figure 3), Middle East, South Africa, South America and Oceania, it is the sole or the most prevalent filarioid species, infesting dogs [9]. Differently from other filarioids transmitted by mosquitoes (e.g., *D. immitis* and *D. repens*) or ticks (e.g., *Cercopithifilaria* spp.) to dogs, *A. reconditum* completes its life cycle in and is vectored by fleas (i.e*., Ctenocephalides canis*, *Ctenocephalides felis*, *Pulex irritans*, *Pulex simulans*, *Echidnophaga gallinae*) or lice (i.e., *Heterodoxus spiniger*, *Linognathus setosus*) with a rate of infestation in fleas of about 5% [9].

Over the last 20 years, *T. callipaeda* has been repeatedly reported to infest the conjunctival sac of domestic (dogs and cats) and wild carnivores (e.g., foxes, wolves, beech martens and wild cats) in Europe [10]. Nowadays, this nematode is recognised as endemic in many European countries (Figure 3) such as France [11,12], Switzerland [13], Spain [14] and Portugal [15] at the similar latitude range (between 39° and 46° N) than those of Asia (between 10° and 45° N for India and Japan, respectively) where the infestation seemed to be confined [16]. Indeed, for its geographical distribution (i.e., in the former Soviet Republics and in many far eastern countries including India, Thailand, China and Japan), this nematode has been known for a long time as “oriental eye-worm” [17]. Since the first evidence of its development in a “bizarre” drosophilid vector, *Phortica variegata* (Diptera, Drosophilidae, Steganinae) [18,19], the occurrence of this helminth seems to be on the rise, probably also due to the improved awareness of parasitologists and practitioners. Interestingly, cases of human thelaziosis in Europe have been diagnosed in north-western Italy, south-eastern France [20] and Spain [21].

*Onchocerca lupi* is an even less known VBH parasitizing the periocular tissues of dogs and cats which has been recognised as a valid species on morphological and molecular grounds [22]. This parasite has been found to infect dogs in southern (Greece, Portugal) and Central Europe (Germany, Hungary, Portugal, Switzerland) [23-27] (Figure 3) and the United States [28-30] where it was recently found also in cats [31]. It causes acute or chronic ocular disease, characterized by conjunctivitis, photophobia, lacrimation, ocular discharge and exophthalmia [32]. Unfortunately, the role played by dogs as reservoirs of *O. lupi* deserves to be assessed and knowledge on the biological vector of this infestation remains meagre [33].

A group of rather neglected filarioids belonging to the genus *Cercopithifilaria* parasitizing the skin of a range of host species [34] has also been recently studied in dogs [35] and three species (namely *Cercopithifilaria* sp.1, *Cercopithifilaria* sp.2 and *Cercopithifilaria grassii*) have been morphologically and molecularly differentiated [36]. In addition, for *Cercopithifilaria* sp.1, the competence of the brown dog tick, *Rhipicephalus sanguineus*, as intermediate host has been experimentally demonstrated [37] and field evidence supports their role as vectors of this filarioid [6]. Following the first retrieval of *Cercopithifilaria* sp.1 from a dog from Sicily, Italy, this filarioid was diagnosed in dogs from Spain, Greece and southern Italy (i.e., Apulia, Basilicata and Sicily regions) (Figure 3), with prevalence rates reaching up to 21.6% [6]. While their pathogenicity to humans is unlikely, there is some evidence indicating the occurrence of skin alterations associated to the presence of larvae in the dermis of dogs [38].

### The role of vectors of VBH in a changing environment

The dissemination of VBH in Europe has been primarily attributed to rapid geographic expansion of their vectors (e.g., invasive mosquitoes, zoophilic fruit flies) and/or increases in their population density. This results from the interaction of several factors, such as the availability of suitable hosts, the arthropod’s adaptability to different environmental conditions, its feeding behaviour and host preferences [39-41]. There have also been extensive debates on the effects of climate change in Europe [42,43], since warmer climates could favour mosquito breeding and, along with higher air temperatures, shorten their extrinsic incubation periods as demonstrated for *Stegomyia aegypti* [*Ae. aegypti*] [44]. Indeed, projected increment in temperature will impact on insect vectors through broadening areas of colonization, invasion of new sites and, eventually, resulting in physiological changes and increased vector capacity. For example, climate change (e.g., increase in mean temperatures) has affected the mosquito abundance and their seasonal survival in many areas of Europe greatly impacting on the spread of filarial infestation [7]. Growing degree-day models (GDD), using wide or local scale temperature data, predicted the occurrence and seasonality of *Dirofilaria* spp. in different parts of the world. These models were based on the minimum threshold of 14°C for the development of *Dirofilaria* in their vector, the requirement of 130 GDD for larvae to reach infectivity and a maximum life expectancy of 30 days for a vector mosquito [7]. Therefore, it was predicted that, due to global warming and raising of mean temperatures, most of the European countries will be suitable for *Dirofilaria* transmission, with a lengthening in the duration of the filarial transmission season [7].

**Figure 4:**
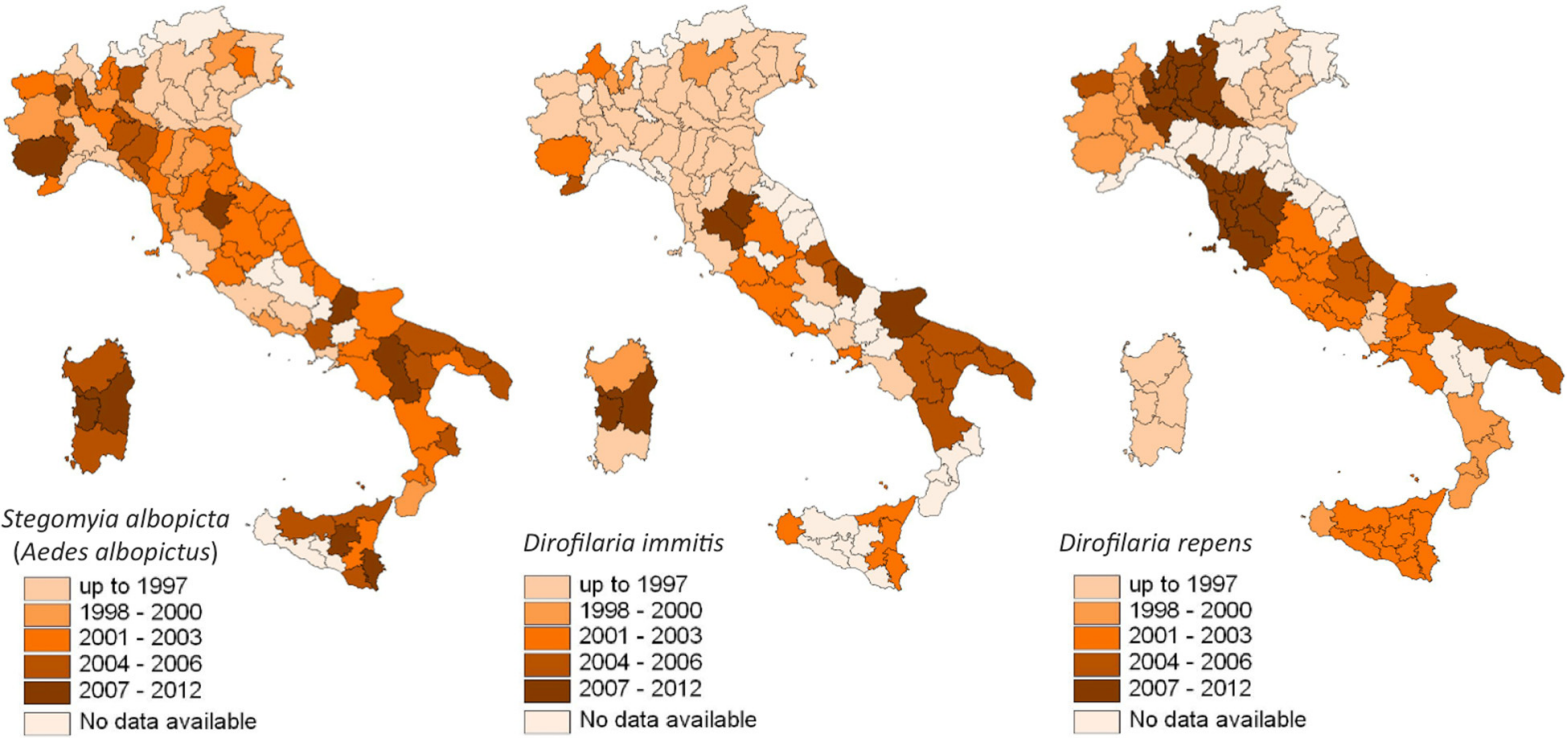
**Distribution of *Stegomyia albopicta*, *Dirofilaria immitis *and *D*. *repens *in Italy.** Map of Italy showing the spread of the distribution of the mosquito *Stegomyia albopicta* [*Ae*. *albopictus*] (left), of *Dirofilaria immitis* in dogs (centre) and *Dirofilaria repens* (right). For *D*. *immitis* and *D. repens* the classes after 1997 correspond to the first report in the region [47,53] and/or an upsurge of the prevalence in dogs compared to the past.

**Figure 5:**
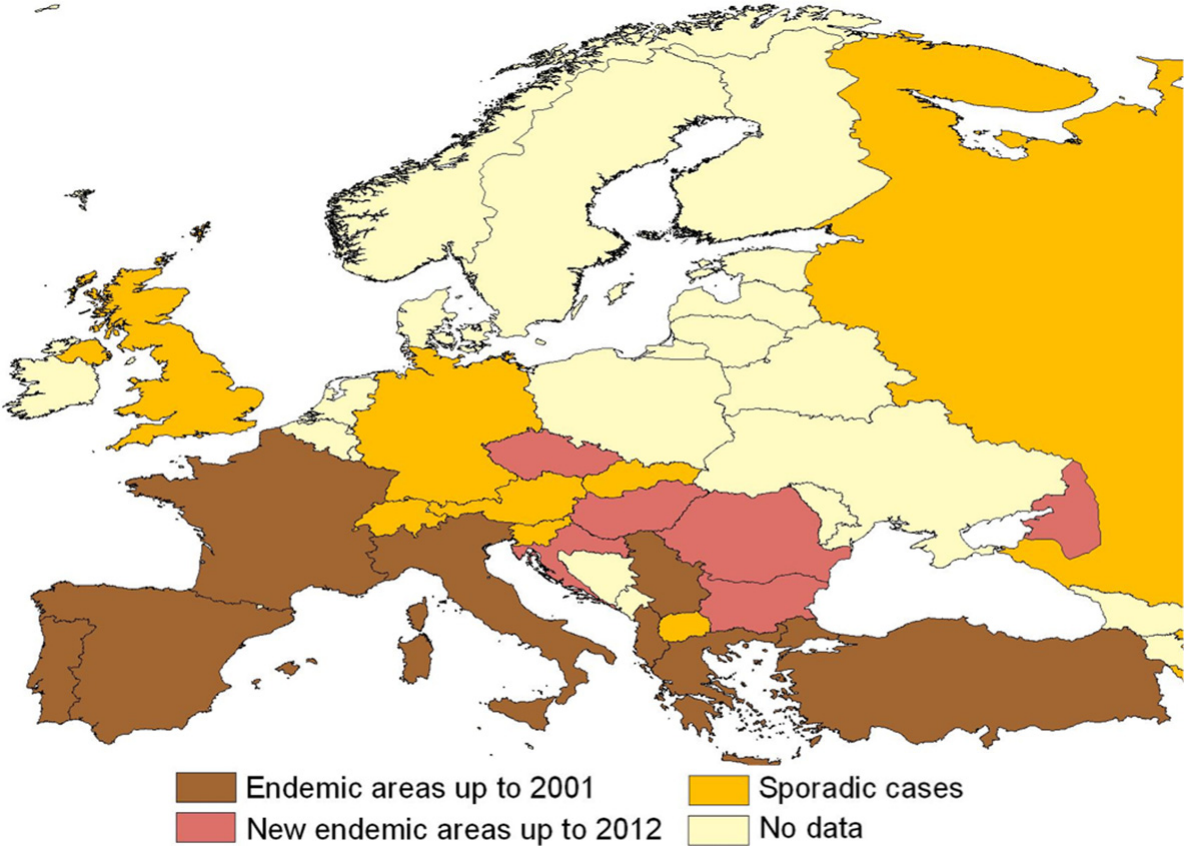
***Dirofilaria immitis *in Europe.** Distribution of *Dirofilaria immitis* in dogs up to 2001 and later on. Modified from [3].

**Figure 6:**
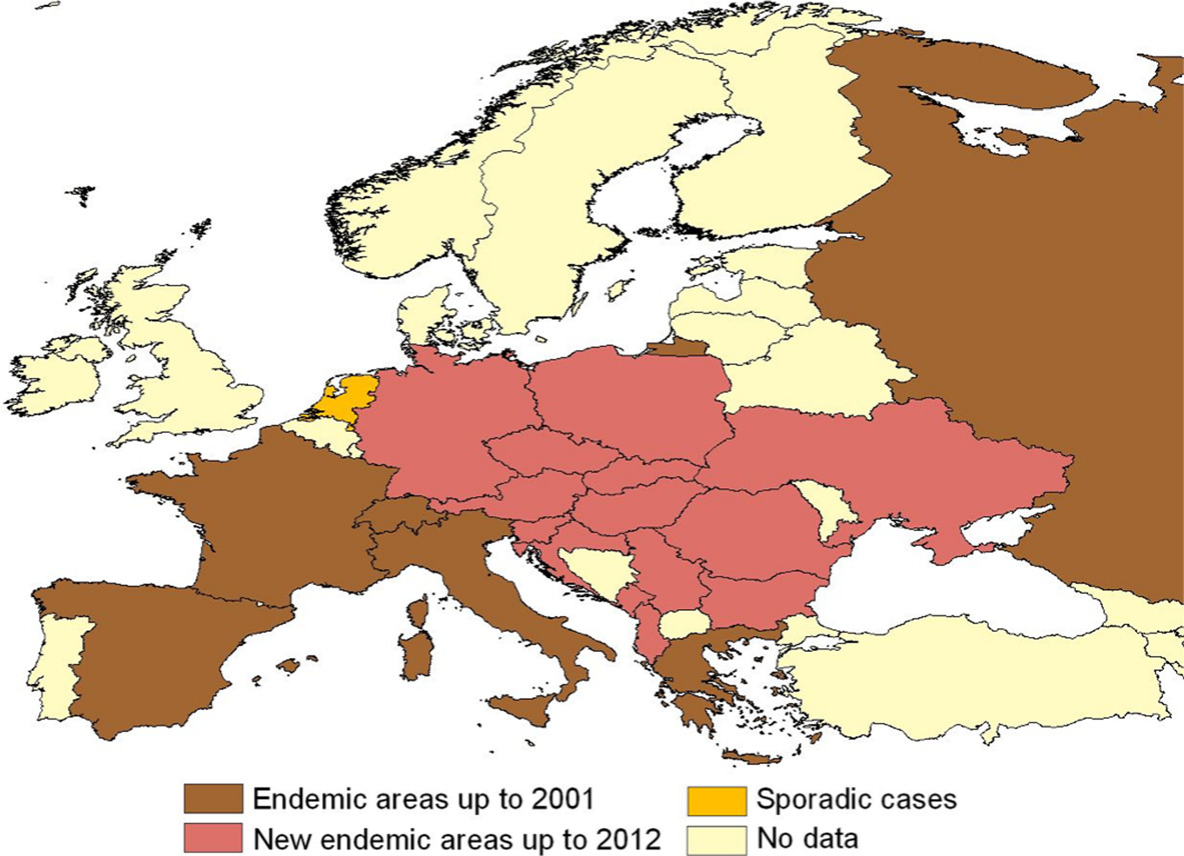
***Dirofilaria repens *in Europe.** Distribution of *Dirofilaria repens* in dogs up to 2001 and later on. Modified from [3].

In addition, several intrinsic factors linked to the specific mosquito vector species also impact on the distribution of VBH. For example, based on retrospective evidence, the expansion of dirofilariosis in Europe somehow matched the second introduction of *St. albopicta* (in 1990 in Italy) [45] but it was not before 2000–2002, when both *D. immitis* and *D. repens* were found in natural populations of Asian tiger mosquito in Italy [46]. Accordingly, the rapid spread of this vector species throughout the country likely broadened the dirofilariosis range to southern regions not previously infected [47] (Figure 4), although the same areas were inhabited by *Cx. p. pipiens*, which is considered the main vector of both *Dirofilaria* in Europe. The sympatric occurrence of both vectors, having diurnal and nocturnal biting activities, may enhance the risk of infestation to dogs and humans, thus increasing the vector-host contact and, eventually, the number of vectors which may carry filarioids in endemic areas throughout the day. Interestingly, over the past decades, *Cx. p. pipiens* has changed its endophagic and antropophagic behaviour in Central and North Europe [4] where actually it also searches for human blood outdoors, as it happens in southern parts of the continent. This pattern also overlaps with the spread of canine *D*. *immitis* and *D. repens* infestation in central and north-eastern countries (e.g. south of Switzerland, Czech Republic, Hungary, Serbia and Slovak Republic) [7,48-52] (Figures 5,6).

Black flies may play a role in the transmission of *O. lupi* in dogs and humans, no convincing scientific evidence in this regard has been produced so far [33]. One of the suspected vectors of *O. lupi*, *Simulium reptans*, is present in areas where the cases of dog ocular onchocerciasis have also been reported (i.e., Portugal, Switzerland, Germany and Hungary) [33]. Until about 50 years ago, *S. reptans* was the dominant black fly in the middle part of Danube valley, whereas today it is extremely rare in this area and it moved southwards down into the Balkan Peninsula [54]. However, in addition biting midges (Diptera, Ceratopogonidae) feeding on a wide range of hosts (humans, livestock and other mammals, amphibians, and birds) might be implicated in the transmission of *O. lupi* since some species of *Culicoides* have been involved in transmission of *Onchocerca cervicalis* and *Onchocerca gutturosa* to horses and cattle, respectively, in Europe [55].

In recent years, the use of the Geographic Information System (GIS) and predictive model algorithms provided important practical contributions to the investigation of the spatial component of the epidemiology of infectious diseases [56], including vector-borne diseases [57,58]. Moreover, the collection of georeferenced epidemiological data can also be useful for disease cluster identification and geostatistical analyses. For example, regional climate model scenarios coupled with high-resolution observations shows that during the 1960–1980s southern France, northern Italy, the northern coast of Spain, the eastern coast of the Adriatic Sea and western Turkey were climatically suitable areas for the establishment of the invasive Asian tiger mosquito, *St. albopicta*. Over the last two decades, climate conditions have become more suitable for the Asian tiger mosquito over Benelux, western Germany and the Balkans, while they have become less suitable over southern Spain. Similar trends are likely to be observed in the future, with an increased risk simulated over northern Europe and slightly decreased risk over southern Europe where drier and warmer summers might limit southward expansion of this species [59]. At the same time, six more indigenous mosquito species, *Culex theileri*[60], *Anopheles maculipennis sensu lato*, *Coquillettidia richiardii*[61], *Aedimorphus vexans*, *Dahliana geniculata* [*Aedes geniculatus*] and *Ochlerotatus caspius*[62-64] have recently been found infected by *D. immitis* in nature. All of the potential indigenous vectors are highly mammophylic and anthropophylic (excluding some members of *An. maculipennis* complex and *Cx. theileri* that only occasionally feed on humans) and could increase transmission rate of these filarioids.

*Phortica variegata*, the vector of *T. callipaeda*, was studied in an area of Italy where dog thelaziosis is highly prevalent [16] and found to be more active at 20–25°C and 50–75% RH during July-August in southern Italy [19]. Suitable environments for the geographic distribution and development of *P. variegata* across Italy and Europe were predicted using a desktop implementation of the Genetic Algorithm for Rule-Set Prediction and all recent reports of *T. callipaeda* fall within the suitable areas indicated by the model [12,14,15,65]. Based on this model, the number of reports of *T*. *callipaeda* infestation may be expected to increase over the next years in areas where it is now considered as non-endemic.

### Host-vector interactions

The availability of suitable hosts for a given VBH and the vector’s feeding behaviour are among the most important factors impacting on VBH distribution. For example, domestic dogs are excellent reservoirs of filarioids, being able to survive for a long time with a considerable worm burden, to harbour different species of filarioids at the same time and to provide infectious microfilariae for competent vectors all over their season of activity. Indeed, among filarioids only *D. immitis* can cause a fatal and severe disease, but presently the majority of dogs harbour a low-medium burden of nematodes, and they show a few symptoms with chronic progression in almost all the cases [66]. Without a doubt, in order to act as good reservoirs of VBH dogs also need to be attractive for competent mosquito vectors as well as tolerant to mosquito bites. For example, in a heartworm endemic north-eastern area of Italy, 70% of *Cx. p. pipiens* and 90% of *Oc. caspius* collected using dog-baited traps from June to September were engorged [67] and it was shown that the number of bites/dog/night can vary according to the vector density, weather conditions and dog size (average of 32.4 and maximum of 81 bites/dog/night). However, the attractiveness and tolerance to mosquitoes of other mammalian species should be considered when studying the potential reservoirs for filarioids in nature. Indeed, dogs are significantly more attractive to eight species of mosquitos (*Aedimorphus taeniorhynchus* [*Aedes taeniorhynchus*], *Culex pipiens quinquefasciatus*, *An. maculipennis*, *Oc. caspius*, *Culiseta annulata*, *Ochlerotatus scapularis*, *Culex declarator* and *Cx. p. pipiens*) than cats [68,69]. In spite of this, cats are not good reservoirs for *D. immitis*, mainly due to host resistance, as inferred by the low adult worm burden in natural and experimental infections, the long prepatent period (8 months), the low level and short duration of microfilaraemia and life span of adult worms (2–3 years) in this host species [70]. Interestingly, some species or strains of mosquitoes displayed inherent mechanisms of defence (refractoriness) to infestation such as the ability of the cibarial armature to destroy microfilariae, the anticoagulant activity of mosquito salivary proteins on the bolus containing microfilariae and other arthropod immunological responses to larvae [71].

Prevalence of microfilaraemic dogs and presence and abundance of competent vectors also affect the rate of infestation within a given mosquito population, which, in turn, is directly related to the risk for a native dog to be infested. In an endemic area of north-eastern Italy out of 40,000 culicids captured from May to October, and screened for *D. immitis* and *D. repens* with a real-time polymerase chain reaction (PCR), *Cx. p. pipiens, Oc. caspius* and *Am. vexans* were found positive for *D. immitis* with an estimated rate of infestation ranging from 0.21 to 1.11%, according to date and site [64]. In the same study, *D. repens* was found in *Cx. p. pipiens* only (rate of infestation of 0.23–0.71%). Interestingly, the rate of infestations did not vary significantly according to season, indicating that in spring a certain number of dogs may be ready to act as reservoirs and thus needing prolonged preventive treatments. In Turkey, an infestation rate of 0.41% and of 0.12% was recorded in *Am. vexans*, the main vector of *D. immitis* in this area, and in *Cx. p. pipiens*, respectively [63]. By combining the rate of infestations with the bites/dog/night numbers above for *Cx. p. pipiens* in north-eastern Italy, it can be speculated that a dog living in an endemic area has a chance to encounter an infected mosquito every 6 nights during the low abundance mosquito period and every 1.2 nights during the high abundance period of the summer. For sure, this calculation does not take into account the presence and the abundance of *St. albopicta*, which is now established in many areas of southern Europe throughout the year [72]. *Stegomyia albopicta*, a vector of *D. immitis*[46,73] and of *D. repens*[74] is active throughout the whole day and year in southern areas, especially in urban habitats. This scenario might be further complicated in the future by the introduction of new invasive mosquito species, such as *Hulecoeteomyia koreica* [*Aedes koreicus*], which is a potential vector of *D. immitis* in Belgium [75] and north-eastern Italy [76]. This species is colonizing colder environments, therefore increasing the possibility to enlarge the area at risk for dirofilariosis in Europe. The factors enhancing the exposure of the host to the vector (i.e., the dog’s size, the age and the outside habitation) may further increase the risk of *D. immitis* infestation [66,77]. Other variables that are reported as risk factors, such as the sex, the length of the coat and dog’s activities (i.e., guard, hunting, stray dogs vs. pet dogs) are likely to be biased by confounding factors, such as male dogs that are used as guard dogs and kept outside day and night.

In Europe, cats were found infected by *D. immitis* mainly in Italy, France and Portugal [3]. In Italy, the prevalence of feline heartworm disease has been approximately estimated as high as 10% of the known prevalence of the infestation in dogs [70]. However, filarioid infestation in cats can occur also in low endemic areas, as reported in central Italy [78]. Due to the very low worm burden usually found in cats, these animals are regarded as “victims” rather than reservoir of *Dirofilaria* spp. On the contrary, red foxes (*Vulpes vulpes*) were found infected by *D. immitis* in Italy, Spain and Bulgaria [3], with prevalence up to 32% in irrigated areas of Spain [79]. In Italy, out of 132 red foxes examined, 25% harboured microfilariae of *D. immitis*, 0.7% of *D. repens*, 15% of *A. reconditum* and 2.3% of *A. dracunculoides*[80]. Other hosts found infected with *D. immitis* are wolves (*Canis lupus*), in Belarus, Italy and Spain [48,81], jackals (*Canis aureus*) in Bulgaria [82] and otters (*Lutra lutra*) in Portugal and Spain [83]. The first European record of *D. immitis* in ferrets (*Mustela putorius putorius*) has also been reported [84], with a particular and aberrant larval migration to the central nervous system. All these hosts are likely to represent an epi-phenomenon of dog infection, with the exception of red foxes, which may act as a wild reservoir of the infection.

In the case of *T*. *callipaeda*, the reasons underlying the steady spread of this nematode throughout many European countries are not clearly understood, but it seems that the same zoonotic strain of *T*. *callipaeda* circulates in the continent within different animal species and humans [18]. The occurrence of very high prevalence of thelaziosis by *T. callipaeda* in foxes (49.3%) as well as in other wild carnivore species (i.e., wolves, beech martens, brown hares, and wild cats) in some areas of southern Italy where canine thelaziosis is highly prevalent (i.e., about 60% of dogs) indicates the status of hyper-endemicity of eyeworm infestation in this area and the primary role foxes play as reservoirs of the infestation [10]. The seasonality and crepuscular activity of *P. variegata* nicely overlaps the behaviour of those wild species hosts*.* The aforementioned ecological considerations are supported by molecular data on the occurrence of a single haplotype (i.e., h1) of *T. callipaeda* among different host species in the study area [18]. The same h1 was found in other European countries, irrespective of the host species from which they were collected [18]. These molecular findings and studies on *P. variegata* indicate a high level of affinity of the nematode for its vector [18,19,85] and low degree of specificity for definitive hosts. These results support the existence of a sylvatic life cycle for *T. callipaeda* and indicate that the infestation is mainly maintained by a large number of wildlife species that, altogether, could play a role in spreading the disease in many previously non endemic areas of Europe [11,13-16,86]. Finally, the high prevalence of eyeworms in dogs and wildlife should represent an alert for human populations considering the difficulties in the differential diagnosis of the infection.

### Impact of VBH on humans

Among the zoonotic filarioids, *D. immitis* and *D*. *repens* probably represent the species more frequently reported in humans where they are detected predominantly in the subcutaneous tissues, pulmonary vessels, testicles and also in the central nervous system, causing a range of clinical manifestations from asymptomatic to, more rarely, fatal syndromes [1,2,66]. In addition, human infestations by *Dirofilaria* spp. often induce nodular lesions, which may be erroneously diagnosed as cancers, hence representing a further challenge to physicians [2]. While *D*. *immitis* is the main agent of human dirofilariosis in the Americas [28,66], *D*. *repens* has been accounted for a long time as the sole species that infest humans in Europe [87,88]. For example, 28 cases of human dirofilariosis from the Old World erroneously attributed to *D. immitis* were reviewed and re-attributed to *D. repens*[88]. However, cases of human dirofilariosis by *D*. *immitis* have been recently described in Italy, Greece and Spain [89-91] and this trend is at an increase in Europe, most likely paralleling the spread of infestation in dogs in central and north-eastern countries (e.g., south of Switzerland, Czech Republic, Hungary, Serbia and Slovak Republic) [5,7,48-52]. Hundreds of cases of human infestation by VBH have been reported worldwide [1], new cases continue to be reported from new geographic areas and it is likely that many more cases occur and are either unrecognized or go unreported. This is the case of *O. lupi*, which has probably been misdiagnosed for a long time with other filarioids localizing in the eyes. Indeed, *O. lupi* has only been suspected to act as a causative agent of infestation in humans until recently [92], when this species has been unambiguously identified morphologically and molecularly in two patients from Turkey and one from Tunisia who exhibited clinical features similar to those of the infestation in dogs [92,93]. Human thelaziosis is a condition described in several areas of the former Soviet Union and Asian continent (e.g., China, Korea, Japan, Indonesia, Thailand, Taiwan and India), predominantly in poor, rural communities with low health and socio-economic standards, particularly where domestic dogs and other animals (e.g., cats and foxes) are heavily affected and live in close contact with humans [94]. The first four cases of human thelaziosis in Europe were diagnosed in patients coming from the north-west of Italy, south-eastern France [20] and Spain [21], where the infestation had been previously reported in dogs, cats and foxes [11,14,16]. The clinical presentation is characterized by mild conjunctivitis, follicular hypertrophy of the conjunctiva, foreign body sensation, epiphora, itchiness, congestion, swelling, hypersensitivity to light, and keratitis.

Considering the lack of awareness for physicians concerning such exotic parasites (e.g., *O. lupi*) and the possibility of misidentifications, the impact of VHD on human populations in Europe, mainly in remote rural areas, is much likely underestimated at present.

### Managing VBH in dogs and humans

#### Prevention

From the picture above it emerges how difficult the prevention and the treatment of VBH in endemic areas may be. This is mostly an issue for dirofilarioses in dogs which, in turn, can be easily prevented with a number of macrocyclic lactones administered in a way to kill *D. immitis* or *D. repens* larvae before they develop into adults in the heart/lungs or the subcutis, respectively. Several molecules are available in chewable tablets, spot on and injectable formulations administered with different protocols (Table 1).

**Table 1:** **Macrocyclic lactones and dosages licensed in different formulations for the prevention of infestations caused by *Dirofilaria immitis *** **(Di) or *Dirofilaria repens *** **(Dr) in dogs**

**Macrociclic lactone**	**Formulation**	**Dosage**	**Claim**
Ivermectin	Tablets/Chewables	6 mcg/kg	Di, Dr
Ivermectin/Praziquantel	Chewables	6 mcg/kg / 5 mg/kg	Di, Dr
Milbemycin oxime*	Tablets	0.5 mg/kg	Di
Moxidectin	Tablets	3 mcg/kg	Di
	Injectable	0.17 mcg/kg	Di, Dr
Moxidectin/Imidacloprid	Spot on	2.5 mg/Kg / 10 mg/kg	Di
Selamectin	Spot on	6 mg/kg	Di

*Tablets containing either praziquantel or lufenuron are also available for chemoprevention at the same dosage (modified from [66]).

Ivermectin is licensed in the Europe to prevent infestations by *D. immitis* and *D. repens*, while spot-on formulations containing moxidectin and selamectin, the oral products containing milbemycin oxime may be further suitable choices for the prevention of *D. immitis*. The injectable long lasting formulation containing moxidectin showed to be effective in controlling *D. immitis* and *D. repens* infestations for a period of 6 months after a single administration [95,96]. The duration of monthly chemoprophylaxis against *D. immitis* (i.e., year round, six months, or only during the vector season) has been debated for long time [97-99]. Current guidelines on management of *D. immitis* infestation in dogs promoted by the European Scientific Counsel on Companion Animal Parasites (ESCCAP) and by the American Heartworm Society (AHS) suggest extending treatment to 7–8 months or even the year round. The rationale for that relies on the occurrence of certain mosquito vectors, such as *St. albopicta*, which may survive in temperate areas as adults even during winter and, at least, for nine months per year [100].

In addition, the use of broad-spectrum drug formulations enhance owner compliance and assist continued control of other helminths [99] and of certain ectoparasites according to the associated molecules (Table 1). Interestingly, the massive use of preventive measures against *D. immitis* infestation showed a decrease in the prevalence of infestation of unprotected dogs living in the same area, through the reduction of reservoir host population. This is the case of some areas of northern Italy where *D. immitis* was regarded as hyper-endemic until 20 years ago, whereas its prevalence decreased over the last decades [47]. Based on this evidence, preventative chemoprophylaxis should be effectively employed also in communities where heartworm prevalence is low or where it is considered emerging [101]. No data is available on the efficacy of macrocyclic lactones against minor species of filarioids (e.g., *A. reconditum*, *A. dracunculoides* and *Cercopithifilaria* spp.) infesting dogs. Consequently, their prevention currently mostly relies on the vector control [37]. Preventing the contact with the fly intermediate host of *T. callipaeda* by the use of bed nets has been recommended for avoiding human infestation [94]. No information is available on the usefulness of any drug as repellents on animals against *P. variegata*.

#### Treatments

The arsenical melarsomine dihydrochloride is the adulticide compound used for the treatment of canine heartworm, associated to confinement of dogs in cages during and for about a month after the treatment period, in order to prevent potentially fatal pulmonary thromboembolism after the death of the heartworms. Melarsomine is usually injected intramuscularly at the dose of 2.5 mg/kg either in a two-step (first intramuscular injection followed by the second 24 hr later) or three-step (first injection followed by the two-dose protocol 4–6 weeks later) regimen. Even though the three-dose scheme is indicated for those animals with a relevant risk of pulmonary thromboembolism post-treatment, this protocol is recommend by the AHS guidelines for the therapy of all infected dogs [101]. Ivermectin may kill adults of *D. immitis* if administered monthly at the preventive dosage of 6–12 μg/kg for not less than 16–30 months [102]. However, the AHS discourages the *extra label* use of macrocyclic lactones as primary adulticides albeit their partial efficacy against microfilariae [101]. In fact, such prolonged treatment period does not prevent from the onset of cardiopulmonary damage in the infected dogs, thus impairing a full clinical recovery [103,104]. The administration of a macrocyclic lactone for up to 6 months before injecting melarsomine can be beneficial in dogs not requiring urgent therapy, because it can reduce parasite burden and permits immature filarioids to reach adulthood at which time they are fully susceptible to adulticide [66,105]. A novel approach for the treatment of cardiopulmonary dirofilariosis is targeting the *Wolbachia* rickettsial endosymbionts. Treatment with tetracyclines has been reported to damage *D. immitis*, even causing death of adult worms [106]. Long-lasting administration of both doxycycline and ivermectin before or in the place of melarsomine injections can eliminate adult worms and also reduce risk of thromboembolism. Therefore, it has been suggested that a combination of doxycycline (10 mg/kg die for 30 days) and ivermectin (6 mcg/kg every 15 days for 6 months) has a potential efficacy, as high as 73%, in the adulticide therapy in dogs infested with *D. immitis*[107,108].

Although other filarioids of dogs, but not *D. immitis*, are considered less clinically relevant, microfilaricide treatment is required to control *D. repens* microfilaria-associated syndromes (e.g., cutaneous erythema and ulcerative pruritic lesions) and to decrease the risk of human and animal infestation in endemic areas. However, only little information is available for the treatment of subcutaneous filariosis by *D. repens*, such as a combination of injectable melarsomine and oral administration of macrocyclic lactones [109]. The use of prolonged selamectin or moxidectin administration in treating dogs infected by *D. repens* is reputed effective [110,111]. The latter molecule also showed a high degree of efficacy in treating *D. repens* infection, including potential ability to kill adults, after a single administration [112-114]. Surgical options usually rely on heartworm removal by the use of flexible alligator forceps with the aid of fluoroscopy- or trans-oesophageal echocardiography- guides, but the success of these procedures may be influenced by several factors [115].

Although minor species of filarioids infesting dogs, (e.g., *A. reconditum*, *A. dracunculoides* and *Cercopithifilaria* spp.), are considered clinically irrelevant, microfilaricide treatment should be always advocated to limit the reservoir function of infected hosts. Though reports on microfilaricide treatment for minor species are scant, evidence suggests that macrocyclic lactones (e.g., ivermectin, selamectin and moxidectin) are effective against patent infestation when administered at the same dosage recommended for *D. immitis*[116,117].

As far as *O. lupi* infestation, surgical removal of the nodules containing the worms remains the only curative treatment for ocular onchocercosis, even thought developmental stages present in periocular tissues and other parts of the body may cause relapses [32]. No specific pharmacological treatments have been reported yet for *O. lupi* infestation in dogs.

Treatment of domestic animals infested by *T. callipaeda* should be carried out and the topic instillation of organophosphates [118] or moxidectin 1% [119] showed to be highly effective. Imidacloprid 10% and moxidectin 2.5% in spot-on formulation was also effective for the control of dog thelaziosis within five (90.47%) to nine (95.23%) days after treatment [120], allowing to overcome problems due to the mechanical removal of parasites or to the restraining of the animals for the local instillation of drugs in the eyes. The administration of an injectable sustained-release formulation of moxidectin and of a monthly treatment with milbemycin oxime provided some seasonal protection against *T. callipaeda* infestation in dogs from an endemic area of northern Italy [121,122]. Such a chemoprophylaxis approach would more likely reduce the prevalence of dog thelaziosis, and therefore the risk for human infestations in endemic areas.

#### Diagnosis

Laboratory diagnosis of infestations caused by *D. immitis*, *D. repens* or *A. reconditum* is achieved through classical detection of circulating microfilariae, parasite antigens and/or by genetic tools. Microfilariae can be identified in the bloodstream of infected animals by microscopic techniques, using the Knott’s test, which is the gold standard method [66]. Blood circulating microfilariae of *D. immitis* should be discriminated from those of other filarioids that do not infest the heart chambers and arteries (i.e. *D. repens* and *A. reconditum*)*.* Key diagnostic features are differences in morphology and size measurements of particular structures. Head of *D. immitis* is slightly tapered, while that of *A. reconditum* and *D. repens* is blunt. The use of fixation in 2% formalin in the Knott’s test can cause a distinctive artefact in the tail of *A. reconditum* and *D. repens* which become curved (“button-hook” or “umbrella” tail), while that of *D. immitis* remains straight [123,124]. Given that the occurrence of such an artefact may vary considerably, the possibility that *A. reconditum* and *D. repens* larvae present a straight tail cannot be ruled out. Hence, the body length becomes discriminatory for the identification at the species level. Indeed, microfilariae of *D. immitis* are 260–340 mm in length and 5–7.5 mm in width, while those of *D. repens* are longer and slightly wider (325–380 × 5–8 mm) and those of *A. reconditum* are smaller and thinner (240–290 × 4.5–5.5 mm) [123,124]. However, microscopic detection of circulating microfilariae may lack in sensitivity. Single-sex infestations, low parasite burdens, immune reactions or past administration of parasiticides with microfilaricidal activity may cause lack of circulating larvae in up to 20–30% of dogs infected by *D. immitis*[66]. An alternative method for diagnosing *D. immitis* infestation in dogs is the use of commercial kits for the detection of antigens released in the blood by adult females. However, some microfilaraemic animals may score negative at these tests for the low worm burden or for the persistence of microfilariae after the death of adult worms [66,112,113]. No similar tests are available for the other filarioids. Other diagnostic tools may include echocardiography, which has high sensitivity although requiring high professional expertise while performing the test [125].

Dermal microfilariae of *O. lupi* and *Cercopithifilaria* spp. can be detected by skin biopsies. Interestingly, it has been demonstrated that microfilariae of the latter species are unevenly distributed on the body of an infected dog with, however, higher frequencies on the interscapular region and on the head, where the tick vectors usually attach [38]. It should also be taken into account that at least three *Cercopithifilaria* spp., two still unidentified at the species level, may infest dogs. However, dermal microfilariae of these three species can be differentiated morphologically based on their length and presence/absence of lateral alae [36].

Recent molecular-based assays have been reported for the unequivocal identification of filarioids, irrespective of their life cycle stage. Ribosomal or mitochondrial DNA sequence fragments of *D. immitis*, *D. repens* and *A. reconditum* may be amplified and analysed with a restriction fragment length polymorphism, specific PCR amplifications or with primers yielding species-specific amplicons [126,127] and their usefulness has been demonstrated in epidemiological and clinical studies [112,128,129]. Recently, a duplex real-time PCR has been assessed for the discrimination between *D. immitis* and *D. repens* and their quantification in blood samples and mosquitoes [130]. A multiplex PCR based on the amplification of a mitochondrial gene of blood-circulating microfilariae of *D. immitis*, *A. reconditum* and *D. repens* and of *Cercopithifilaria* spp. has also been shown to be useful for their molecular detection and differentiation in blood and skin samples [131]. Finally, a multicentre study in the Mediterranean area proved that the three species of *Cercopithifilaria* affecting dogs might be discriminated from each other by differences in mitochondrial *cox*1 and ribosomal 12S sequences [36].

## Conclusions

Although great scientific achievements have been gained over the past decades on several aspects of the biology, epidemiology, control and treatment for many VBH (e.g., *D. immitis* and *D. repens*), most of them have only been recently known to science, thus they remain enigmatic in many ways. The increasing trend of VBH in Europe is most likely due to the spreading process of several arthropod vector species and highlights the need for actions focussing on control of the vectors in the environment and the protection of animals at individual and population levels. Indeed public health authorities should be concerned about the potential risk of introduction and establishment of new and exotic vectors, which may alter the VBH scenario in a manner that may not be easily foreseeable.

The biological mechanisms behind the increased number of cases of VBH in Europe remain uncertain and research on the role played by insect vectors for many of them is lacking. This is only partially due to the complexity of the relationship between pathogen, host and vector. Indeed, some VBH are relatively poorly investigated and this lack of awareness represents a major constraint to their successful management and control in endemic and non-endemic areas. For example, veterinary/medical surveillance accompanied by entomological surveillance is essential to prevent the spread of filaroids and to evaluate the risk of zoonotic filariosis outbreaks. There is a need to monitor closely the changing epidemiology of VBH in order to predict their future distribution, particularly in light of their constant spread and of socio-economic and political events. Under the above circumstances, the economic crisis and the subsequent population movements from southern to central and northern European areas may render it difficult to afford effective therapeutic treatments and a correct management of the environment, towards the reduction of arthropod vector breeding sites.

In addition, although basic and applied research in the biology of insect vectors is often considered the 'Cinderella' in the political agenda of governmental funding agencies, these are essential for controlling arthropods and VBH, especially given the introduction of European directives which limit the number of available biocides (e.g. 98/8/EC) and ban the aerial use of insecticides (e.g., P6-TA- (2009) 0010) within the European Union. Since the range of alternatives is limited, identifying novel control strategies is essential.

## Competing interests

The authors declare that there are no competing interests.

## Authors’ contributions

DO conceived the review and wrote the first draft of the manuscript with GC, DP, FD-T, EB, CG, DT. All authors equally contributed with the revision of the manuscript. EB elaborated figures. All authors read and approved the final version of the manuscript.
